# Transition-metal-induced magnetism in particles of CaYAl_3_O_7_: Ni, Fe and Co

**DOI:** 10.1039/d6na00181e

**Published:** 2026-05-18

**Authors:** José Laurentino, Luiza Paffer, José Araújo, Francisco Estrada, José Holanda

**Affiliations:** a Programa de Pós-Graduação Em Engenharia Física, Universidade Federal Rural de Pernambuco 54518-430 Cabo de Santo Agostinho Pernambuco Brazil joseholanda.silvajunior@ufrpe.br; b Group of Optoelectronics and Spintronics, Universidade Federal Rural de Pernambuco 54518-430 Cabo de Santo Agostinho Pernambuco Brazil; c Unidade Acadêmica Do Cabo de Santo Agostinho, Universidade Federal Rural de Pernambuco 54518-430 Cabo de Santo Agostinho Pernambuco Brazil; d Facultad de Biologia, Universidad Michoacana de San Nicolas de Hidalgo, Av. F. J. Mujica s/n Cd. Universitaria Morelia Michoacian Mexico; e Programa de Pós-Graduação Em Física Aplicada, Universidade Federal Rural de Pernambuco 52171-900 Recife Pernambuco Brazil; f Programa de Pós Graduação Em Tecnologias Energéticas e Nucleares (Proten), Universidade Federal de Pernambuco 50740-545 Recife PE Brazil

## Abstract

Here, we report the first observation of transition-metal-induced magnetism in CaYAl_3_O_7_ particles doped with Ni, Fe, and Co ions synthesized by the Pechini method. Structural characterization by X-ray diffraction followed by Rietveld refinement confirmed preservation of the tetragonal melilite phase without detectable secondary phases. Microstructural parameters such as crystallite size, lattice strain, and dislocation density were evaluated, revealing only minor structural distortions after doping. Raman spectroscopy further confirmed the structural integrity of the host lattice while indicating local symmetry perturbations caused by dopant incorporation. Magnetic measurements performed by vibrating sample magnetometry revealed clear hysteresis loops with finite coercivity and remanent magnetization, following the trend Co < Fe < Ni. The observed magnetic behavior is attributed to exchange interactions among localized magnetic moments introduced by the transition-metal ions. These results demonstrate that doped CaYAl_3_O_7_ is a promising multifunctional material combining optical and magnetic functionalities for applications in sensing, photonics, and magneto-optic devices.

## Abstract 2

This study presents the first investigation of the magnetic behavior of CaYAl_3_O_7_ particles doped with transition-metal ions (Ni, Fe, and Co).

## Graphical abstract

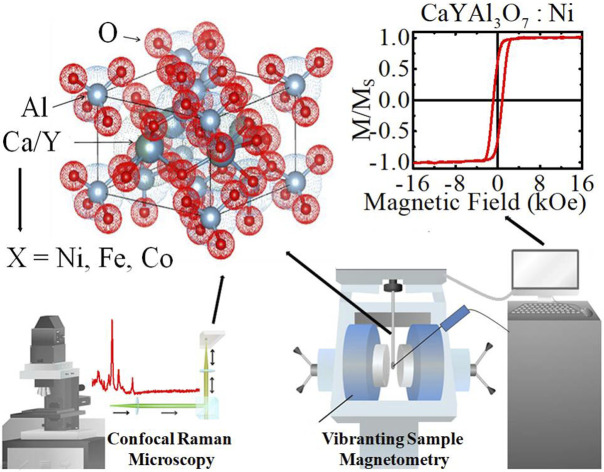

Optical and magnetic properties of particles play a central role in advancing a wide range of technological applications. The ability to control and tailor these characteristics is crucial for the development of innovative materials and devices.^1–3^ Research into the optical and magnetic behavior of materials has significantly expanded our understanding of complex phenomena that are not yet fully understood.^4–6^ In this context, materials doped with rare-earth ions and transition metals are particularly promising, as doping can markedly modify intrinsic properties and impart desirable optical and magnetic functionalities.^7–9^ Among such materials, the host matrix ABC_3_O_7_ stands out due to its exceptional properties. This compound crystallizes in the tetragonal system with space group *P*4_2_1*m*, where A = Ca, Sr, or Ba; B=La or Gd; and C=Al or Ga. Specifically, calcium yttrium aluminate (CaYAl_3_O_7_, CYAM) belongs to the non-centrosymmetric melilite family and has attracted considerable interest owing to its favorable physicochemical stability, availability of raw materials, and straightforward synthesis routes. In addition, CYAM exhibits remarkable luminescent performance when doped or co-doped with rare-earth ions^10,11^ and displays piezoelectric behavior under high-temperature conditions.^12,13^ These attributes make CYAM a strong candidate for photonic applications such as temperature sensing, white-light LED fabrication, and electronic devices including actuators, ultrasonic transducers, and electrical transformers.^7,8,14^

The luminescent properties of CYAM are commonly investigated using undoped powder samples,^14^ which typically exhibit well-defined particle morphologies. Photoluminescence (PL) emission and excitation spectra obtained using synchrotron radiation in the ultraviolet and vacuum-ultraviolet regions indicate a bandgap energy of approximately 6.8 eV or slightly lower.^4–14^ A low-intensity emission band centered near 4.4 eV, observed under excitation at 6.5 eV, is attributed to self-trapped excitons generated during vacuum-ultraviolet excitation. Furthermore, three dominant emission bands peaking at 2.57, 2.94, and 3.23 eV account for the majority of PL and radioluminescence (RL) emissions in CYAM.^11^ Calcium yttrium aluminate phosphors doped with rare-earth ions have been extensively explored to achieve full-color emission, particularly in the red and blue spectral regions.^15–20^ In yttrium-based aluminate systems, emission bands are typically observed within the energy range of 2.53–3.49 eV.^21,22^ Excitonic luminescence generally consists of multiple bands whose spectral positions depend on several factors, including the host bandgap, cation distribution among crystallographic sites, synthesis methodology, and measurement temperature.^22–24^ Although CYAM has been widely studied for its optical properties, investigations focusing on transition-metal doping remain scarce.

Although CaYAl_3_O_7_ has been extensively investigated as a luminescent and piezoelectric host material, its magnetic properties remain virtually unexplored. This lack of knowledge limits the potential use of CaYAl_3_O_7_ in multifunctional devices where optical and magnetic responses are simultaneously required. Therefore, the central problem addressed in this work is whether transition-metal doping can induce intrinsic magnetic ordering in CaYAl_3_O_7_ while preserving its crystalline framework. Demonstrating such coexistence would open new routes toward multifunctional oxide materials. A schematic diagram of the CaYAl_3_O_7_ unit cell, illustrating the substitution of Ca/Y ions by Ni, Fe, or Co ions, is shown in Fig. 1(a). Fig. 1(b) and (c) present the experimental techniques employed in this work: confocal Raman microscopy and vibrating sample magnetometry, respectively. The synthesis of materials with precisely controlled particle size, shape, composition, and properties enables the development of advanced functional systems tailored for specific applications.^1–3,7–17^ Among the available synthesis routes, the Pechini method is particularly attractive due to its ability to produce homogeneous particles with controlled composition and efficient incorporation of dopant ions.^25–32^

**Fig. 1:**
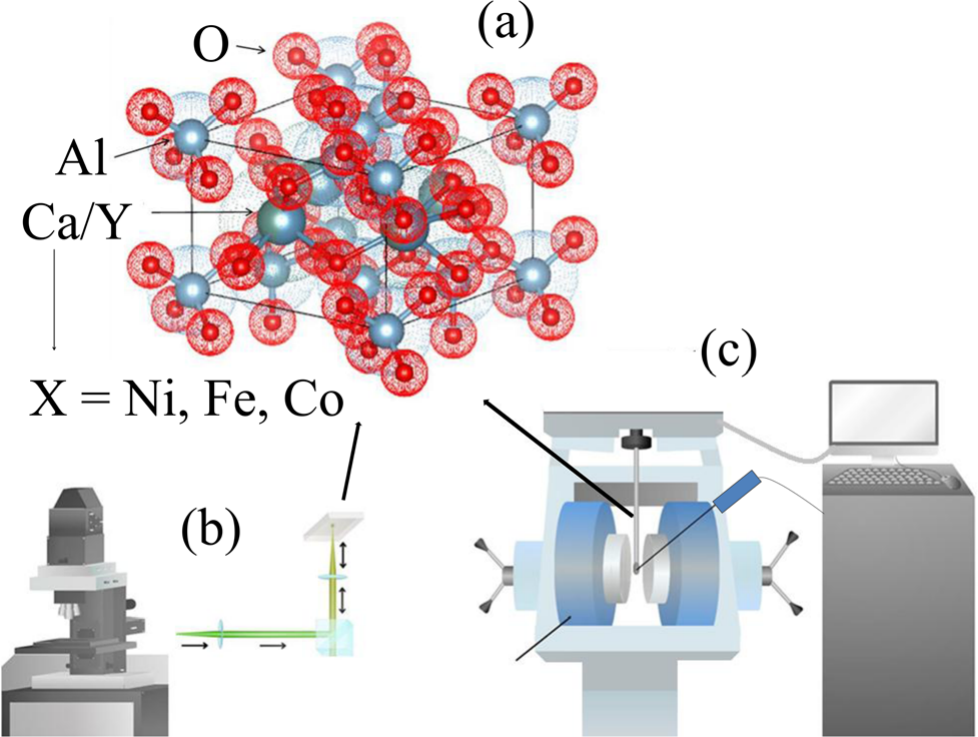
(a) The unit cell structure of CaYAl_3_O_7_, where Ca and Y ions are replaced by Co, Fe, or Ni ions. Sketches of the (b) Confocal Raman Microscopy, and (c) vibrating sample magnetometry techniques during the measurements in CaYAl_3_O_7_: Ni, Fe and Co.

All doped samples were synthesized with a nominal concentration of 10 mol% transition-metal ions (Ni, Fe, or Co). Energy-dispersive X-ray spectroscopy (EDX) elemental analysis confirmed the presence of the corresponding dopants in all samples, with measured concentrations close to the nominal values, indicating successful incorporation during synthesis, as shown in Table 1. The small systematic reduction in lattice parameters is consistent with substitution by smaller transition-metal ions.

**Table 1:** Nominal dopant concentration and EDX-measured transition-metal content for Ni-, Fe-, and Co-doped CaYAl_3_O_7_ samples, confirming successful incorporation and reliable compositional control during synthesis

Sample	Nominal dopant (%)	EDX measured (%)
CaYAl_3_O_7_ : Ni	10	9.7 ± 0.5
CaYAl_3_O_7_ : Fe	10	10.2 ± 0.4
CaYAl_3_O_7_ : Co	10	9.8 ± 0.6

The incorporation efficiency and structural distortion induced by transition-metal ions strongly depend on ionic radius mismatch relative to host cation sites. For octahedral coordination, the ionic radii are approximately Ni^2+^ = 0.69 Å, Fe^3+^ = 0.645 Å, and Co^2+^ = 0.745 Å, values comparable to Ca^2+^ and Y^3+^ substitutional environments. Such size compatibility favors lattice incorporation while generating local strain fields capable of modifying optical and magnetic interactions. Similar ionic-radius-controlled behavior has been reported in recent oxide systems.^33,34^Fig. 2(a) shows the X-ray diffraction (XRD) patterns of undoped CaYAl_3_O_7_ and samples doped with Ni, Fe, and Co. All diffraction peaks match well with the reference pattern of CaYAl_3_O_7_ (ICSD #09438),^7–14^ confirming the preservation of the tetragonal melilite structure after transition-metal doping and indicating the absence of secondary phases within the detection limit. An enlarged view of the main diffraction region reveals similar peak positions and relative intensities for all samples, suggesting that the incorporation of Ni, Fe, and Co does not significantly distort the host lattice.^7–12^ As shown in Fig. 2(b), TEM micrographs reveal polycrystalline particles with average sizes ranging from 45 to 70 nm, in good agreement with the XRD crystallite size estimates. No segregated nanoparticles or secondary phases were observed, further supporting the phase purity of the synthesized samples.

**Fig. 2:**
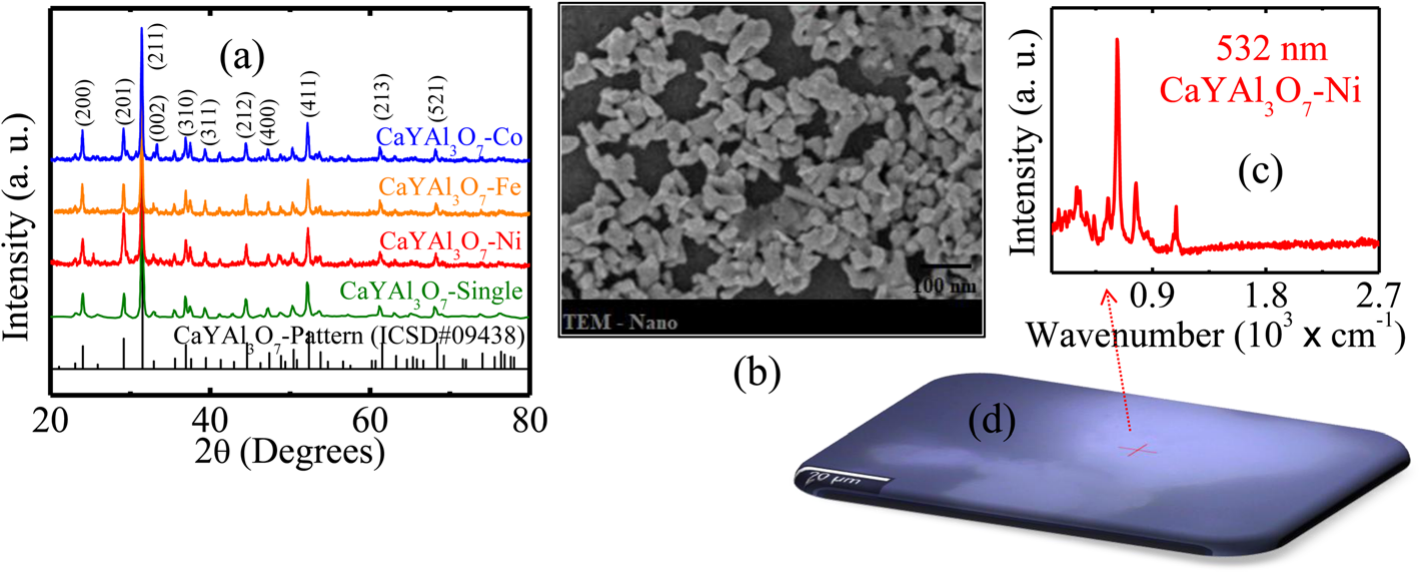
(a) X-ray diffraction measurements of pure CaYAl_3_O_7_, and doped with Ni, Fe, and Co samples, compared with the ICSD#09438 pattern. (b) TEM micrograph showing polycrystalline particles with average sizes ranging from 45 to 70 nm. Spectrum (c) and Raman map (d) of the sample CaYAl_3_O_7_ : Ni excited with a 532 nm laser.

The average crystallite size (*D*) was estimated using the Scherrer equation, and the dislocation density (*δ*) was calculated using *δ* = 1/*D*^2^. The obtained values are summarized in Table 2. All doped samples exhibited only slight increases in dislocation density compared with undoped CaYAl_3_O_7_, indicating that transition-metal incorporation generates limited crystallographic defects while preserving structural stability. Rietveld refinement confirmed that all samples crystallize in the tetragonal melilite structure (space group *P*4_2_1*m*). No secondary crystalline phases associated with NiO, Fe_2_O_3_, CoO, or cobalt ferrites were detected within the instrumental resolution. Small variations in lattice parameters were observed, consistent with substitutional incorporation of dopant ions, as shown in Table 3.

**Table 2:** Average crystallite size (*D*), estimated from the Scherrer equation, and corresponding dislocation density (*δ*) for pure and transition-metal-doped CaYAl_3_O_7_ samples obtained from XRD refinement data

Sample	Crystallite size (nm)	Dislocation density (×10^15^ lines per m^2^)
Pure CYAO	60	0.27
Ni-doped	57	0.31
Fe-doped	55	0.33
Co-doped	53	0.36

**Table 3:** Lattice parameters (*a* and *c*) and refinement quality factor (*χ*^2^) obtained from Rietveld analysis of pure, Ni-, Fe-, and Co-doped CaYAl_3_O_7_ samples. All compositions were indexed in the tetragonal melilite structure with space group *P*4_2_1*m*

Sample	*a* (Å)	*c* (Å)	*χ* ^2^
Pure	7.214	4.918	1.31
Ni	7.209	4.915	1.28
Fe	7.206	4.912	1.34
Co	7.203	4.909	1.36

Fig. 2(c) and (d) present the Raman mapping and Raman spectrum, respectively, of CaYAl_3_O_7_ : Ni excited with a 532 nm laser. The observed vibrational modes are characteristic of the melilite structure and further confirm the structural integrity of the host matrix upon doping. Raman spectroscopy probes inelastic light scattering induced by lattice vibrations, enabling identification of chemical composition and local structural changes.^7–14^ Furthermore, Raman measurements were performed using excitation wavelengths of 532 nm and 633 nm, with particles deposited on a microscope slide. Fig. 3 presents the Raman spectra of CaYAl_3_O_7_ particles doped with transition metals, measured using excitation wavelengths of 532 nm and 633 nm. Panels Fig. 3 (a) and (b) show the spectra of CaYAl_3_O_7_ : Ni obtained with 633 nm and 532 nm excitation, respectively. Panels Fig. 3 (c) and (d) present the Raman spectra of CaYAl_3_O_7_ : Fe measured at 532 nm and 633 nm.

**Fig. 3:**
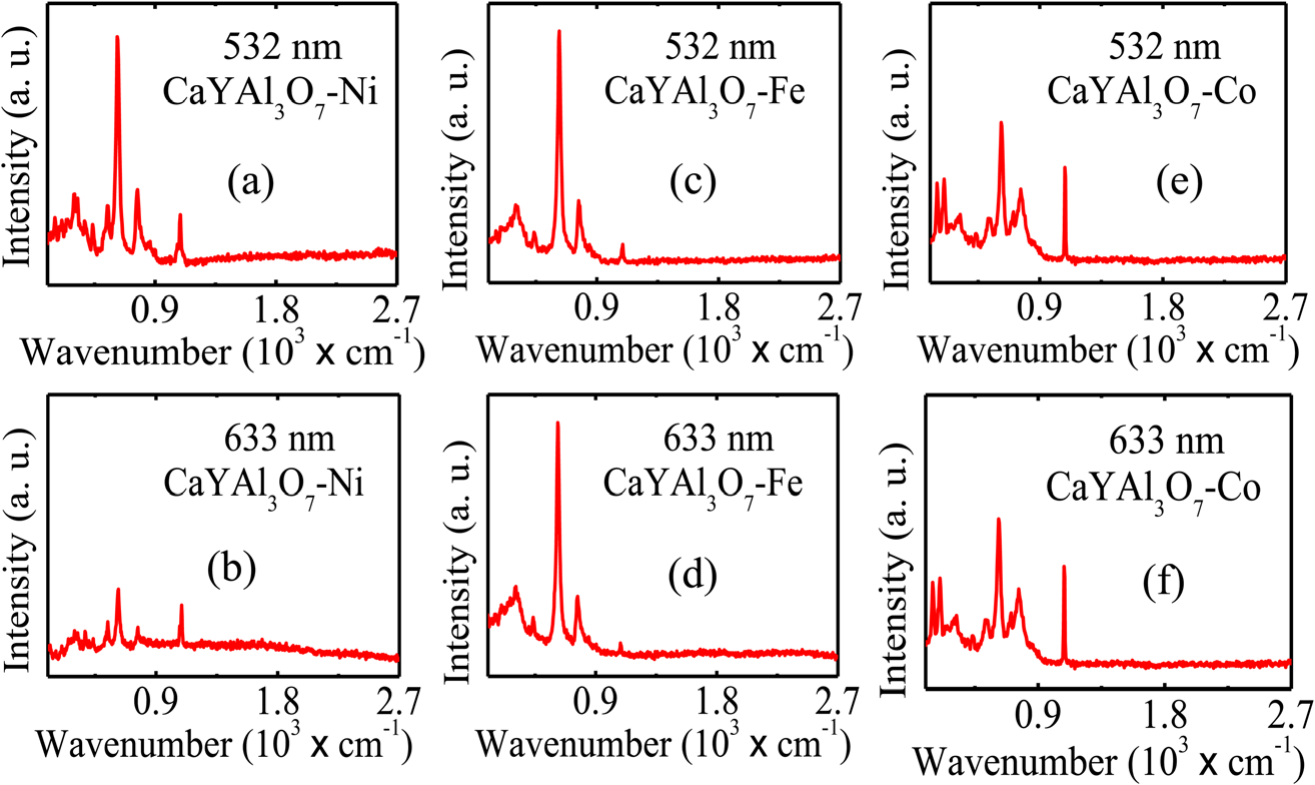
Raman spectra of CaYAl_3_O_7_ particles doped with transition metals, measured using excitation wavelengths of 532 nm and 633 nm. Panels (a) and (b) show the spectra of CaYAl_3_O_7_ : Ni obtained with 633 nm and 532 nm excitation, respectively. Panels (c) and (d) present the Raman spectra of CaYAl_3_O_7_ : Fe measured with 532 nm and 633 nm excitation. Panels (e) and (f) correspond to CaYAl_3_O_7_ : Co acquired using 532 nm and 633 nm excitation, respectively. The dominant Raman modes observed below ∼900 cm^−1^ are characteristic of the tetragonal melilite-type CaYAl_3_O_7_ structure and are primarily associated with Al–O bending and stretching vibrations of the [AlO_4_] tetrahedra. No additional Raman-active modes related to secondary phases are detected. Variations in peak intensity, linewidth, and resonance behavior with dopant type and excitation wavelength indicate local lattice distortions and modified electron-phonon coupling induced by the incorporation of Ni, Fe, and Co ions, while preserving the host crystal symmetry.

Panels Fig. 3 (e) and (f) correspond to CaYAl_3_O_7_ : Co acquired with 532 nm and 633 nm excitation, respectively. The observed Raman modes in the range 0.4–2.7 × 10^3^ cm^−1^ are characteristic of the melilite-type CaYAl_3_O_7_ structure and are primarily associated with Al–O vibrational modes of the [AlO_4_] tetrahedra. Variations in peak intensity and linewidth with dopant type and excitation wavelength indicate modifications of the local lattice environment induced by the incorporation of Ni, Fe, and Co ions, while preserving the host crystal symmetry. No additional Raman-active modes associated with secondary phases of Ni, Fe, or Co are observed. All Raman peaks correspond to vibrational modes of the CaYAl_3_O_7_ melilite lattice, primarily associated with Al–O vibrations of the [AlO_4_] tetrahedra. Dopant incorporation leads to variations in peak intensity, linewidth, and resonance behavior, indicating local lattice distortions and modified electron–phonon coupling without altering the host crystal symmetry.^30–32^

While CaYAl_3_O_7_ retains its excellent optical properties upon transition-metal doping, we further investigated whether these doped materials exhibit magnetic behavior. Remarkably, magnetic hysteresis loops were clearly observed, as shown in Fig. 4. The doped samples exhibit finite coercivity and remanent magnetization, following the trend CaYAl_3_O_7_ : Co < CaYAl_3_O_7_ : Fe < CaYAl_3_O_7_ : Ni, as shown in Fig. 4(a)–(c). The strength of the magnetic response is governed by the electronic configuration and magnetic moment of the transition-metal ions, as well as the exchange interactions between localized spins incorporated into the CaYAl_3_O_7_ lattice. The successful incorporation of Ni, Fe, and Co ions enhances spin–spin interactions within the host matrix, producing magnetic dipole moments sufficiently strong to be detected through hysteresis measurements. Magnetic characterization was performed using vibrating sample magnetometry, as illustrated in Fig. 1(c).

**Fig. 4:**
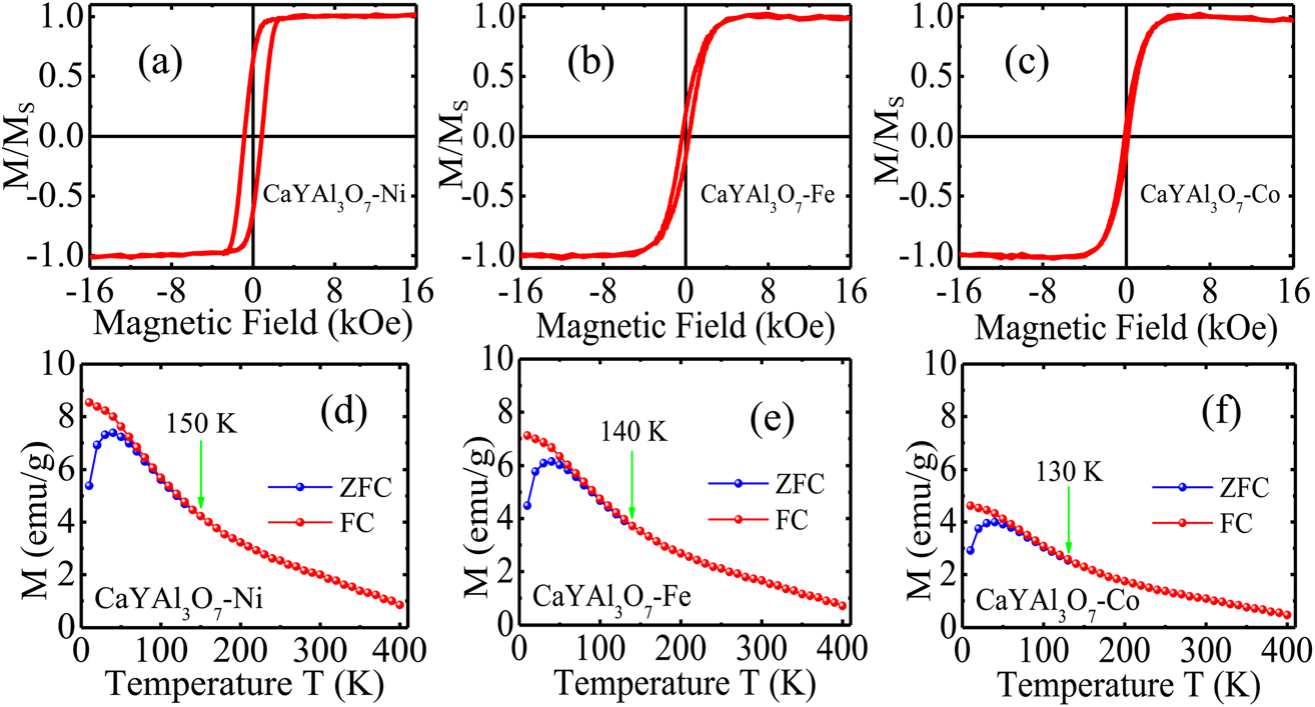
Magnetic hysteresis loops measured by vibrating sample magnetometry (VSM) for CaYAl_3_O_7_ particles doped with transition metals: (a) CaYAl_3_O_7_ : Ni, (b) CaYAl_3_O_7_ : Fe, and (c) CaYAl_3_O_7_ : Co. The presence of finite coercivity and remanent magnetization in all samples indicates the emergence of magnetic behavior induced by transition-metal doping. Temperature-dependent magnetization (*M*–*T*) curves measured under ZFC and FC (*H* = 90 Oe) conditions, showing clear and persistent magnetic ordering above room temperature, with a gradual decrease in magnetization as temperature increases. No sharp magnetic transition is observed below 400 K, indicating strong and thermally stable magnetic coupling. A weak ZFC/FC bifurcation suggests additional contributions from nanoscale anisotropy and defect-mediated magnetic interactions: (d) CaYAl_3_O_7_ : Ni, (e) CaYAl_3_O_7_ : Fe, and (f) CaYAl_3_O_7_ : Co.

The observed magnetic behavior is attributed predominantly to intrinsic exchange interactions among localized magnetic moments introduced by substitutional Ni^2+^, Fe^2+^, and Co^2+^ ions occupying Ca/Y lattice positions, as shown in the data in Table 4. The absence of secondary phases in Rietveld refinement and TEM strongly supports this interpretation. Additionally, oxygen vacancies and local lattice distortions generated during aliovalent substitution may contribute to defect-mediated ferromagnetic coupling, as reported in diluted magnetic oxides. Therefore, the magnetism arises from a combined mechanism involving direct superexchange and defect-assisted interactions. The values presented in Table 4 confirm the magnetic trend Co < Fe < Ni, which is associated with progressively stronger magnetic moments and enhanced exchange coupling.^5,32^

**Table 4:** Saturation magnetization (*M*_S_), remanent magnetization (*m*_r_), and coercive field (*H*_C_) obtained from VSM measurements for transition-metal-doped CaYAl_3_O_7_ samples

Sample	*M*_S_ (emu per g)	*m*_r_ (emu per g)	*H*_C_ (Oe)
Ni-doped	3.104	1.945	845
Fe-doped	7.219	1.618	325
Co-doped	8.360	1.321	206

Fig. 4 (d)–(f) presents the magnetization–temperature (*M*–*T*) curves measured under zero-field-cooled (ZFC) and field-cooled (FC) conditions at 90 Oe. All samples exhibit clear and persistent magnetic ordering well above room temperature, with magnetization decreasing only gradually as the temperature increases, as shown in Fig. 4(d)–(f). No abrupt magnetic transition is observed below 400 K, evidencing strong and thermally stable magnetic coupling. Furthermore, the weak bifurcation between the ZFC and FC curves suggests additional contributions from nanoscale anisotropy and magnetic interactions. These results strongly reinforce the presence of robust intrinsic room-temperature ferromagnetism in the doped samples.

The successful incorporation of Ni, Fe, and Co ions enhances exchange-mediated spin–spin interactions within the host matrix, generating localized magnetic moments detectable through hysteresis measurements, as commonly observed in diluted magnetic oxides and transition-metal-doped ceramics.^33–36^ In conclusion, this work demonstrates for the first time that transition-metal doping transforms CaYAl_3_O_7_ from a purely optical host into a multifunctional magnetic material. Structural analyses confirmed phase stability after Ni, Fe, and Co incorporation, while Raman spectroscopy revealed only local lattice perturbations. Magnetic hysteresis loops with finite coercivity and remanence proved the emergence of intrinsic magnetic behavior, strongest for Co doping. These findings establish doped CaYAl_3_O_7_ as a new platform for magneto-optical devices, sensors, and multifunctional ceramic technologies.

## Author contributions

J. L., L. P., J. A., F. E. analyzed all the experimental measures and J. H. discussed, wrote and supervised the work.

## Conflicts of interest

The authors declare that they have no conflict of interest.

## Data Availability

Data will be made available on reasonable request.
